# Immunomodulatory Effects of LSI312A on Dendritic Cells: A Novel Approach to Modulating Inflammatory Pathways

**DOI:** 10.4014/jmb.2506.06027

**Published:** 2025-08-18

**Authors:** Hien Thi Thu Do, Chaelin Lee, Inmoo Rhee

**Affiliations:** Department of Biotechnology and Bioscience, Sejong University, Seoul 05006, Republic of Korea

**Keywords:** Homoisoflavonoid, dendritic cells, inflammation

## Abstract

Inflammation plays a crucial role in the pathogenesis of various diseases, necessitating the development of effective anti-inflammatory therapeutics. Dendritic cells (DCs), as professional antigen-presenting cells, are key regulators of immune responses. In this study, we investigated the immunomodulatory effects of LSI312A, a novel compound derived from medicinal plant analogues, on DC function and inflammatory signaling pathways. LSI312A exhibited no cytotoxicity in DC2.4 cells at concentrations up to 20 μM. LSI312A significantly reduced antigen uptake and impaired the expression of co-stimulatory molecules, particularly MHC class II and CD40, upon lipopolysaccharide (LPS) stimulation. Moreover, LSI312A markedly suppressed the secretion of pro-inflammatory cytokines, including TNF-α and IL-6, and decreased nitric oxide (NO) production by downregulating iNOS expression at both the mRNA and protein levels. Mechanistically, LSI312A inhibited the phosphorylation of NF-κB, a central regulator of inflammatory responses, while promoting Nrf2 nuclear translocation, an essential factor in antioxidant signaling. Furthermore, LSI312A effectively suppressed the activation of the PI3K/Akt pathway, contributing to its anti-inflammatory effects. These results suggest that LSI312A modulates key inflammatory pathways and DC-mediated immune responses, highlighting its potential as a novel therapeutic candidate for inflammation-related diseases.

## Introduction

Inflammation is a critical biological response to injury, infection, and other stressors, playing a central role in the pathogenesis of various diseases, including autoimmune disorders, cancer, and cardiovascular diseases [1-3]. While acute inflammation is essential for the body's defense mechanisms, chronic inflammation can contribute to tissue damage and disease progression [4]. Therefore, the development of effective anti-inflammatory therapeutics remains a major challenge in modern medicine [5, 6].

Dendritic cells (DCs), as professional antigen-presenting cells, are pivotal in initiating and regulating immune responses [7, 8]. They capture, process, and present antigens to T cells, thereby influencing the direction of the immune system’s response [9]. DCs are not only critical for the induction of adaptive immunity but also serve as a bridge between the innate and adaptive immune systems [10]. Upon activation by pathogen-associated molecular patterns (PAMPs), such as lipopolysaccharide (LPS), DCs undergo phenotypic and functional changes that include increased antigen uptake, co-stimulatory molecule expression (*e.g.*, MHC class II and CD40), and the secretion of pro-inflammatory cytokines like TNF-α and IL-6 [11-15]. These responses are tightly regulated by key intracellular signaling pathways, including the NF-κB and PI3K/Akt pathways, which mediate inflammation and immune activation [16, 17]. However, dysregulation of these pathways can lead to excessive inflammation and immune-related disorders [18, 19].

Cremastranone, a homoisoflavonoid with antiangiogenic activity, has been extracted and purified from various plants, including *Chionodoxa luciliae* and *Cremastra appendiculata* [20, 21]. The first total synthesis of natural cremastranone derivatives was achieved in three to five steps using key transformations such as selective 1,4-reduction, aldol condensation, and targeted demethylation [22]. This synthetic approach provides access to diverse homoisoflavonoid analogues, enabling further studies on their biological activities and structure-activity relationships.

Given the central role of DCs in immune regulation, modulating their function presents a promising strategy for therapeutic intervention in inflammatory diseases. LSI312A, a homoisoflavonoid cremastranone analogues, has emerged as a potential anti-inflammatory agent. Previous studies have suggested its ability to influence various cellular functions, yet its impact on DC function and the underlying molecular mechanisms remain largely unexplored. In this study, we investigate the immunomodulatory effects of LSI312A on DC-mediated immune responses, focusing on its ability to regulate antigen uptake, co-stimulatory molecule expression, cytokine secretion, and the activation of key inflammatory signaling pathways. Our findings provide valuable insights into the potential of LSI312A as a therapeutic candidate for inflammation-related diseases.

## Materials and Methods

### Chemical and Cell Culture

Cell culture medium includes RPMI 1640 (Capricorn, Cat No. RPMI-A, Germany), fetal bovine serum (FBS)(Corning, Cat No. 35-015-CV, US), penicillin-streptomycin (Gibco, Cat No.15140-122, USA), L-glutamine (Gibco, Cat No. 25030-081). Murine dendritic cell 2.4 (DC2.4) were cultured in RPMI 1640, respectively, supplemented with 10% FBS, penicillin-streptomycin, especially adding L-glutamine to DC 2.4 culture medium. LSI312A (formerly synthesized as SH15004), an antiangiogenic homoisoflavonoid, is a synthetic compound based on the active component derived from herbal extracts of *C. luciliae* and *C. appendiculata*. Its first total synthesis was achieved in 3–5 steps using selective 1,4-reduction, aldol condensation, and demethylation, with contributions from Dr. Seung-Yong Seo of Gachon University [22].

### Cell Viability Assay

DC 2.4 cells (4 × 10^5^ cells per well) were seeded into a 24-well plate. After 24 h incubation in the absence or presence of LSI312A (1, 5, 10, 20 μM), all cells were collected and washed in 2% FBS in PBS. The cell viability was assessed by APC annexin V apoptosis detection kit with 7-AAD (Biolegend, Cat No. 640930, USA). Briefly, cells were resuspended in Annexin V binding buffer and stained with APC-Annexin V and 7-ADD viability staining solution for 15 min in an ice box, and finally analyzed with BD BioScience FACSCalibur cytometer, and the cell viability was calculated.

### Nitric Oxide Production Assay

DC 2.4 cells (4 × 10^5^ cells per well) were seeded into a 24-well plate and treated with a concentration array of LPS-EK (Invivogen, Cat No. tlrl-eklps, USA). The cells were stimulated with the presence or absence of the drug LSI312A (1, 5, 10, 20 μM) for 16 h, then the supernatant was collected and divided into 96-well plates. NO concentration was detected by Griess reagents with Griess reagent A compounding 2% phosphoric acid H_3_PO_4_ and 1% sulfanilamide (Sigma-Aldrich, Cat No. S9251, USA), and Griess reagent B consisting of N-(1-Naphthyl) ethylenediamine Dihydrochloride (Tokyo Chemical Industry Co., Cat No. N0063, Japan). The supernatant is respectively mixed with Griess reagent A, and Griess reagent B following a ten-minute interval without the light. The absorbance was measured at 540 nm by Spectramax M2 microplate reader and the concentration of NO was calculated following the standard curve.

### Cell Stimulation and Cytokine Detection

The cells (5 × 10^4^ cells per well) were plated in a 96-well plate and treated with the indicated concentration of LPS and LSI312A for 24 h. Cytokine production (TNF-α, IL-6) was measured in cell supernatant with TNF-α and IL-6 ELISA kit such as DuoSet Mouse TNF-α (R&D systems, Cat No. DY410, US) and DuoSet Mouse IL-6 (R&D systems, Cat No. DY406, US) according to the manufacturer’s instructions. The reactions were stopped by H_2_SO_4_ (Junsei, Cat No. 83010S0350, Japan), and the absorbance was detected at 450 nm. The assays were performed in triplicate.

### Western Blotting

2.5 × 10^6^ DC 2.4 cells were subjected to the indicated formulation in a 6-well plate for 20 h and lysed with a cold lysis buffer, such as TNE buffer containing 50 mM Tris-HCl (Tris - Duchefa Biochemie, Cat No. 77-86-1, HCl -Samchun, Cat No. 7647-01-0, Republic of Korea), 2 mM EDTA (Sigma-Aldrich, E8008, USA), 1% NP-40 (Millipore, Cat No. 492016, USA), or RIPA buffer (Millipore, Cat No. 20-288, USA) together with Protease Inhibitor cocktail (Roche, Cat No. 11836170001, Germany) and Phosphatase Inhibitor cocktail (Roche, Cat No. 04906837001, Germany). Lysate was cleared by centrifugation for 10 min at 4°C. The collected lysates were electrophoresed through SDS-PAGE and then transferred into PVDF blotting membrane (GE Healthcare, Cat No. 1060002, USA). After blocking for 30 min, the membrane was incubated overnight at 4°C or 1 h at room temperature (R.T) with the primary antibody. The membrane was then incubated in the HRP-conjugated goat anti-mouse IgG or goat anti-rabbit IgG followed by detection using enhanced chemiluminescence (ECL) (Bio-Rad, Cat No. 1705061, USA). The blots were measured via ImageJ and the ratios were compared with the β-actin blots.

For cytoplasmic and nuclear extraction, the cells were lysed in TNE buffer for 10 min, then centrifuged at 16,400 ×*g*, 4°C for 10 min. The lysates was collected as cytoplasmic extraction, the left pellet was suspended in RIPA buffer for 40 min with a vortex 10-min interval. After centrifuging at 16,400 ×*g*, 4°C for 10 min, the supernatant was transferred into another tube, referring as nuclear extraction. Both cytoplasmic and nuclear extraction were loaded to the SDS-PAGE gel and transferred to PVDF membranes. These membranes were stained with the antibody to Nrf2, NF-κB, and β-actin followed by the described detection.

The primary antibodies: iNOS (BD Bioscience, Cat No. 610329, 1:500, USA), β-actin (Santa Cruz, Cat No. sc-47778, 1:500, USA), phospho-NF-κB (Santa Cruz, Cat No. sc-136548, 1:500), NF-κB (Santa Cruz, Cat No. sc-8008, 1:200), Nrf2 (Cell signaling, Cat No. 12721, 1:1000, USA), Rabbit Akt Ab (Cell Signaling, Cat#4691), Rabbit phospho-Akt Ab (Cell Signaling, Cat#4060), corresponding horseradish peroxidase (HRP)-conjugated goat anti-mouse IgG (Jackson ImmunoResearch Laboratories, Cat No. 115-036-003, 1:10000, USA), goat anti-rabbit IgG (Bio-Rad, Cat No. 170-6515, 1:10000, USA).

### Intracellular Reactive Oxygen Species Production Assay

DC 2.4 cells (2.5 × 10^6^ cell/well) were treated with the indicated treatment in a 6-well plate for 20 h, then harvested and washed by DPBS. The clean cells were stained with a cell-permeable dye H_2_DCFDA (Invitrogen, Cat No. D399, 200 μM, USA) for 30 min. After gentle washing, the cells were resuspended in DPBS (Welgene, Cat No. 001-02, Republic of Korea) and the fluorescent intensity was measured by BD BioScience FACsCalibur cytometer.

### Real-Time Quantitative PCR

Total RNA was extracted using Trizol reagent (Invitrogen, Cat No. 15596026, USA) following the manufacturer’s recommendation. cDNA was synthesized by reverse transcription using RevertAid first strand cDNA synthesis kit (Thermo Fisher Scientific, Cat No. K1622, USA) and quantitative real-time PCR was performed using PowerSYBR Green PCR Master Mix (Applied Biosystems, Cat No. 4367659, USA) in StepOneTM real-time PCR system (Thermo Fisher Scientific). Data were analyzed with the 2^(-ΔCT)^ method. Data were normalized based on β-actin determined in the same sample. Analysis of all samples was performed in triplicate. Primers were synthesized by Cosmo Genetech company.

### Statistical Analysis

All experiments were repeated with at least triplicate assays. Statistical analysis was performed using GraphPad Prism 5 software. A paired, two-tailed Student’s *t*-test or two-way ANOVA was used to compare groups. *p* values < 0.05 were considered significant.

## Results

### Effect of LSI312A on Antigen-Presenting Ability of Dendritic Cells

To assess the potential cytotoxic effects of LSI312A on dendritic cells, we treated DC2.4 cells with increasing concentrations of LSI312A. Cell viability was determined by assessing cell growth, and the results indicated no significant cytotoxicity at concentrations up to 20 μM, suggesting that LSI312A does not adversely affect cell survival under the experimental conditions (Fig. S1). Based on these findings, concentrations up to 20 μM were used in all subsequent experiments to investigate the immunomodulatory effects of LSI312A on dendritic cell function.

Dendritic cells (DCs) are essential in antigen presentation and the initiation of adaptive immune responses [23]. A key function of DCs is their ability to take up and process antigens, which are then presented to T cells to activate the immune response [24]. To evaluate how LSI312A affects the antigen uptake capacity of DCs, we treated DC2.4 cells with lipopolysaccharide (LPS) to activate them, in combination with LSI312A (10 μM). The cells were incubated with FITC-labeled antigens, either FITC-Dextran or FITC-BSA, and antigen uptake was quantified by flow cytometry.

**Fig. 1:**
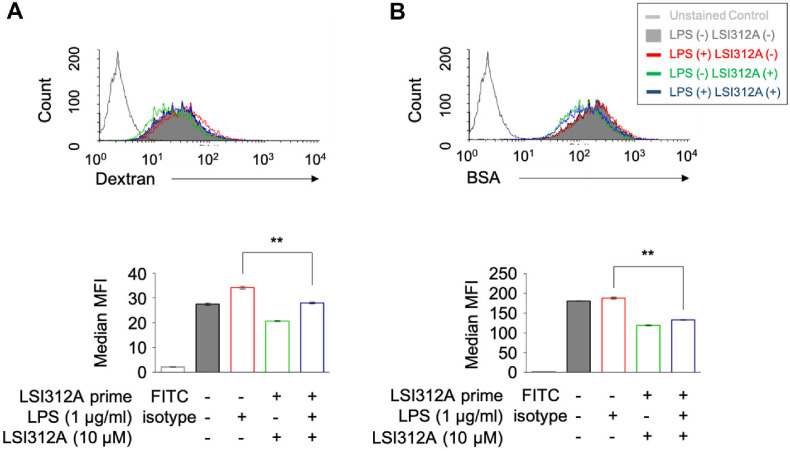
The impairing activity of LSI312A on the antigen uptake of dendritic cells. Dendritic cells (DC2.4 cells) were primed with LSI312A for 16h. The next day cells were treated with LPS (1 μg/ml) and LSI312A (10 μM) for 1 h, then supplied by FITC-Dextran (100 mg/ml) or FITC-BSA (10 mg/ml) for 4 h. The cells were collected to analyze by flow cytometry to determine the Ag amount taken up by DCs. Representative histograms are shown, and the mean fluorescent intensity (MFI) is displayed as the mean ± SEM of values from three distinct experiments. Statistical differences were analyzed by a two-tailed, paired *t*-test, **p < 0.01, as compared with the LSI312A-untreated group.

The results clearly demonstrated that LSI312A significantly reduced the uptake of both FITC-Dextran and FITC-BSA by DC2.4 cells (Fig. 1). This reduction in antigen uptake suggests that LSI312A may impair the early stages of antigen processing in DCs, which could hinder the activation of T cells. The diminished ability of DCs to internalize antigens in the presence of LSI312A indicates that it interferes with the normal functioning of these immune cells, potentially impacting the initiation of adaptive immune responses.

**Fig. 2:**
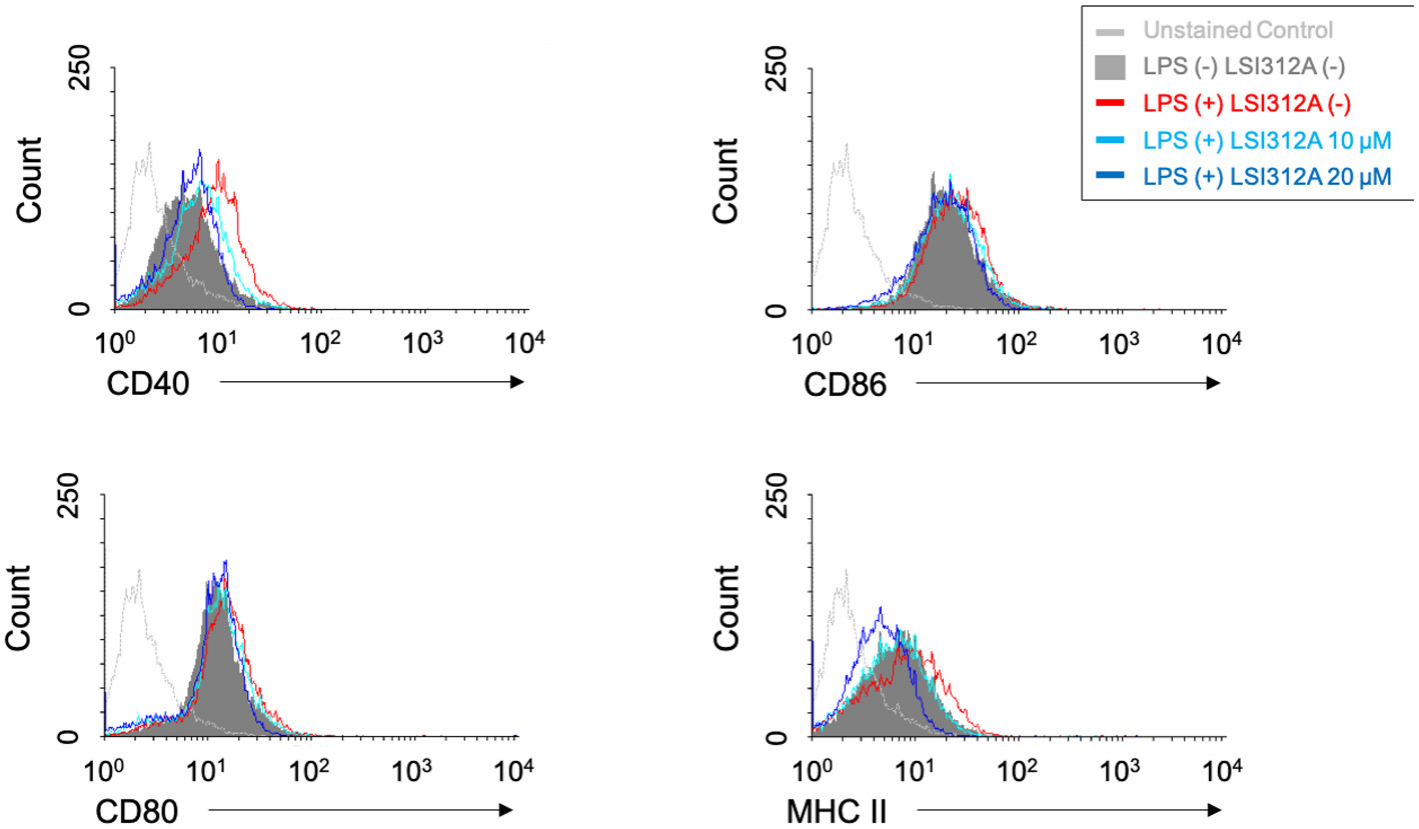
The effect of LSI312A on the expression of co-stimulatory molecules and MHC class II on dendritic cells. DC2.4 cells were activated by LPS (1 μg/ml) with or without the stimulation of LSI312A (10 and 20 μM) for 20 h. The cells were harvested and stained with fluorescent conjugated Ab for CD40, CD80, CD86, and MHC class II. The expression of membrane proteins was analyzed by flow cytometry. Representative histograms are shown and the mean fluorescent intensity (MFI) is displayed as the mean ± SEM of values from three distinct experiments. Statistical differences were analyzed by a twotailed, paired *t*-test, *p < 0.05, as compared with the LSI312A-untreated group.

Next, we examined the expression of co-stimulatory molecules and MHC class II, both of which are essential for effective antigen presentation [25]. After 20 h of LPS and LSI312A treatment, flow cytometry analysis revealed that LPS stimulation significantly increased the surface expression of CD40, CD80, CD86, and MHC class II on DC2.4 cells, which is expected upon DC activation. However, LSI312A treatment significantly downregulated the expression of MHC class II and CD40 (Fig. 2). These co-stimulatory molecules are critical for the interaction of DCs with CD4^+^ T cells, and their decreased expression suggests that LSI312A may modulate DC activation status and potentially affect their capacity to stimulate T cells.

### Inhibitory Effect of LSI312A on Pro-Inflammatory Cytokine Production

Cytokine secretion by dendritic cells plays a critical role in mediating inflammatory responses and recruiting other immune cells to the site of infection or injury [26]. To evaluate the impact of LSI312A on cytokine production, DC2.4 cells were treated with LPS to activate the inflammatory response and subsequently treated with LSI312A (5, 10 μM). The levels of pro-inflammatory cytokines TNF-α and IL-6 were measured in the culture supernatant by ELISA.

**Fig. 3:**
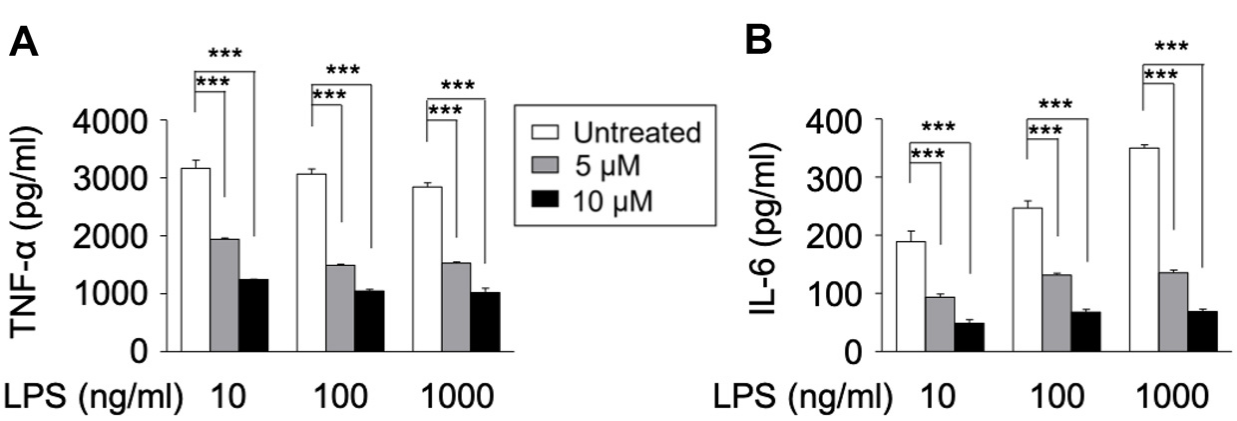
The decrease of cytokine production by LSI312A treatment. DC2.4 cells were activated by LPS and LSI312A drug for 24 h. The cell medium was harvested for cytokine ELISA assay to detect the secretion of TNF-α, IL-6. Results are presented as the mean ± SEM of values obtained from triplicate cultures. Statistical differences were analyzed by two-way ANOVA, ***p < 0.001, as compared with the LSI312A-untreated group.

Our results showed that LSI312A treatment significantly suppressed the release of TNF-α and IL-6 in LPS-stimulated DCs (Fig. 3). TNF-α and IL-6 are key mediators of inflammation and contribute to the activation and recruitment of other immune cells, such as macrophages and neutrophils. The observed reduction in their secretion indicates that LSI312A attenuates pro-inflammatory cytokine production in dendritic cells, suggesting its potential to dampen excessive inflammatory responses.

### Suppression of Nitric Oxide Production by LSI312A

Nitric oxide (NO) is an important inflammatory mediator produced by immune cells, including dendritic cells [27]. NO is involved in vasodilation, immune cell recruitment, and pathogen elimination [28]. However, excessive NO production can contribute to tissue damage and exacerbate inflammation [29]. To determine the effect of LSI312A on NO production, we measured the levels of NO in the culture supernatant of LPS-stimulated DC2.4 cells in the presence or absence of LSI312A.

**Fig. 4:**
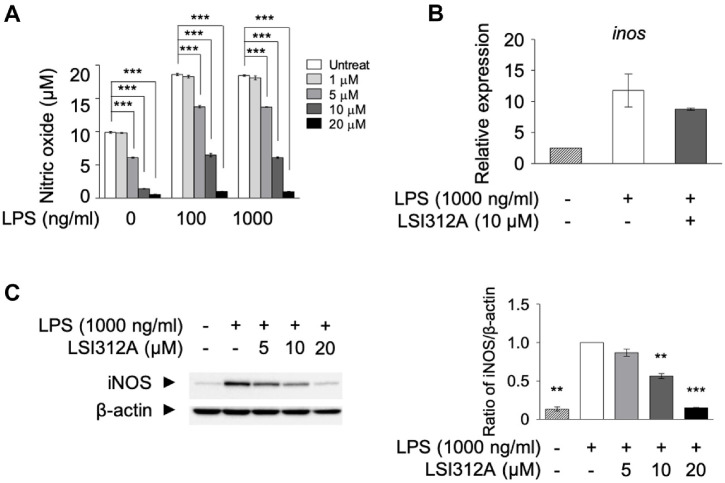
The inhibitory effect of LSI312A on the production of nitric oxide in dendritic cells. (**A**) DC 2.4 cells were stimulated with various combinations of LPS (100, 1000 ng/ml) and LSI312A (1, 5, 10, and 20 μM) for 16 h. Nitric oxide production was measured in the supernatant. (**B**) DC 2.4 cells were treated with LPS (1 μg/ml) and LSI312A (10 μM) for 16 h. RNA was isolated, cDNA was synthesized, and quantitative PCR was performed using SYBR Green. (**C**) DC 2.4 cells were activated with LPS (1,000 ng/ml) and LSI312A in a dose-dependent manner for 20 h. iNOS expression was measured by western blotting. The blots were quantified using ImageJ, and results were normalized to β-actin. Results are presented as mean ± SEM from triplicate samples. Statistical analysis was performed using two-way ANOVA (**p < 0.01, ***p < 0.001) compared to the LSI312A-untreated group.

The results revealed that LSI312A treatment led to a dose-dependent reduction in NO production, with significant decreases observed at concentrations of 10 and 20 μM (Fig. 4A). This finding indicates that LSI312A suppresses NO production in dendritic cells, which contributes to attenuating inflammatory responses. To further explore the mechanism underlying this effect, we analyzed the expression of inducible nitric oxide synthase (iNOS), the enzyme responsible for NO production, at both the mRNA and protein levels. Real-time quantitative PCR (qPCR) analysis showed that LPS stimulation significantly increased inos gene transcription after 16 h, while LSI312A treatment resulted in a marked reduction (Fig. 4B). Consistently, Western blot analysis demonstrated that LSI312A also decreased iNOS protein expression (Fig. 4C). These results suggest that LSI312A inhibits NO production by downregulating iNOS expression in LPS-stimulated dendritic cells.

### Inhibition of NF-κB Activation by LSI312A

**Fig. 5:**
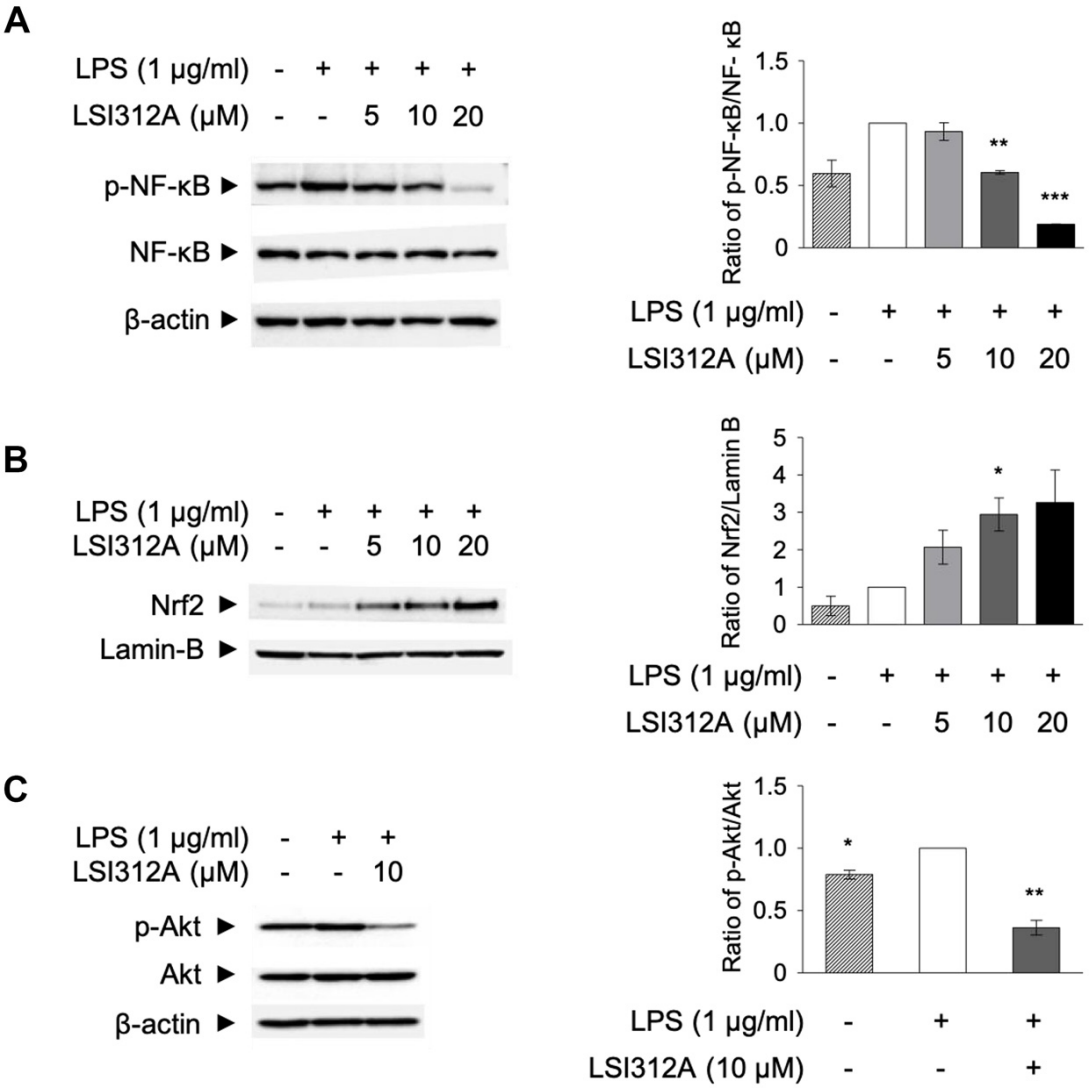
The modulation of LSI312A on signaling pathway. DC 2.4 cells were treated with LSI312A in a dose-dependent manner, along with LPS (1,000 ng/ml). (**A**) After 20 h, cells were collected in TNE buffer, and the supernatant was analyzed for NF-κB phosphorylation by western blotting. (**B**) After 2 h, cells were collected for cytoplasmic extraction, and the pellets were used for nuclear extraction by resuspending them in RIPA buffer. Nrf2 expression was measured in the nuclear fraction using an anti-Nrf2 antibody. (**C**) After 20 h, cells were harvested in TNE buffer, and Akt phosphorylation was detected by western blotting. The results were quantified using ImageJ and normalized to β-actin or LaminB. Experiments were repeated three times, and data are presented as mean ± SEM. Statistical analysis was done using a two-tailed paired *t*-test, with *p < 0.05 and **p < 0.01 compared to the untreated LSI312A group.

The NF-κB signaling pathway is a key regulator of inflammation, immune responses, and antigen presentation [30]. Activation of NF-κB occurs in response to various stimuli, including pathogen recognition by pattern recognition receptors (PRRs) such as TLRs [31]. To assess whether LSI312A modulates NF-κB activation, we measured its phosphorylation status in LPS-treated DC2.4 cells. Western blot analysis showed that LPS stimulation significantly increased NF-κB phosphorylation compared to unstimulated controls. Treatment with LSI312A resulted in a concentration-dependent reduction in NF-κB phosphorylation levels (Fig. 5A).

While decreased phosphorylation suggests that LSI312A may suppress NF-κB signaling activity, it is important to note that phosphorylation alone does not confirm functional inhibition of the pathway. Further studies assessing nuclear translocation of NF-κB and the expression of downstream target genes are required to validate this potential inhibitory effect.

### Promotion of Nrf2 Nuclear Translocation by LSI312A

The Nrf2 pathway plays a critical role in regulating the cellular response to oxidative stress and inflammation [32]. Upon activation, Nrf2 translocates to the nucleus and promotes the transcription of genes involved in antioxidant defense, thereby protecting cells from oxidative damage [33]. To assess whether LSI312A influences the Nrf2 pathway, we examined Nrf2 nuclear translocation in LPS-stimulated DC2.4 cells treated with LSI312A.

Analysis of nuclear and cytoplasmic fractions showed that LPS stimulation alone did not significantly alter Nrf2 nuclear translocation compared to controls. Treatment with LSI312A resulted in a significant increase in nuclear Nrf2 expression (Fig. 5B). We would like to clarify that these data represent Nrf2 levels in the nuclear fraction, not total cellular protein. While increased nuclear Nrf2 suggests potential activation of antioxidant signaling pathways, it is important to note that without assessment of downstream target gene expression, such as HO-1 or NQO1 induction, definitive conclusions regarding functional antioxidant activation cannot be made. Further studies are needed to confirm the biological significance of this observation.

### Modulation of Akt Phosphorylation by LSI312A

The PI3K/Akt pathway plays an essential role in regulating cell survival, growth, and inflammation [34]. It has also been shown to modulate NF-κB activation, and its dysregulation is associated with various inflammatory and immune-related diseases [35]. To assess whether LSI312A influences the PI3K/Akt pathway, we examined Akt phosphorylation in LPS-treated DC2.4 cells following LSI312A treatment.

Western blot analysis revealed that LPS stimulation increased Akt phosphorylation compared to unstimulated controls, and treatment with LSI312A led to a reduction in Akt phosphorylation levels (Fig. 5C). While this decrease suggests that LSI312A may suppress Akt pathway activation, further studies are required to determine the functional consequences of Akt modulation, including effects on cell survival and inflammatory gene expression.

## Discussion

In this study, we investigated the immunomodulatory effects of LSI312A on DC function and inflammatory signaling pathways. Our results demonstrate that LSI312A significantly impairs antigen uptake, downregulates the expression of co-stimulatory molecules such as CD40 and MHC class II, and suppresses the secretion of key pro-inflammatory cytokines including TNF-α and IL-6 [36-39]. Additionally, LSI312A inhibited nitric oxide production and modulated critical intracellular signaling pathways associated with DC activation. To our knowledge, this is the first report examining the effects of LSI312A on dendritic cells. A recent in-press study demonstrated that LSI312A suppressed myeloid-derived suppressor cell (MDSC) activation and reduced inflammatory cytokine production, suggesting that its immunomodulatory actions extend across multiple innate immune cell types. This cross-lineage effect may be particularly relevant in inflammatory diseases where MDSCs and DCs coordinate immune responses.

Previous studies on small-molecule natural compounds such as honokiol and quercetin have shown similar immunomodulatory effects in dendritic cells. For instance, honokiol has been reported to inhibit LPS-induced upregulation of co-stimulatory molecules and cytokine production in bone marrow-derived dendritic cells by suppressing NF-κB activation [44, 45]. Likewise, quercetin was shown to attenuate DC-mediated inflammatory responses by inhibiting PI3K/Akt and NF-κB pathways, leading to reduced antigen presentation and T cell activation. Our findings with LSI312A are consistent with these studies, indicating that suppression of NF-κB and modulation of related signaling pathways are common mechanisms underlying small-molecule inhibition of DC activation.

Mechanistically, NF-κB is a central transcription factor regulating the expression of various pro-inflammatory genes, including cytokines and co-stimulatory molecules. The inhibition of NF-κB phosphorylation by LSI312A likely contributes to the observed reduction in dendritic cell activation markers and cytokine secretion. While our Western blot results demonstrate decreased NF-κB protein levels, the absence of nuclear fractionation and downstream gene expression analysis limits our ability to confirm direct transcriptional inhibition. Future studies incorporating nuclear translocation assays and analysis of NF-κB target genes will be necessary to validate these mechanistic interpretations.

The observed increase in Nrf2 protein levels following LSI312A treatment suggests a potential role in activating antioxidant defense pathways. Nrf2 regulates the transcription of antioxidant enzymes such as heme oxygenase-1 (HO-1) and NAD(P)H:quinone oxidoreductase 1 (NQO1), which play crucial roles in counteracting oxidative stress and resolving inflammation [46, 47]. Prior studies have shown that pharmacological activation of Nrf2 in dendritic cells can promote an anti-inflammatory phenotype. However, our data demonstrate only an increase in total Nrf2 protein levels without confirmation of nuclear translocation or downstream gene induction. Therefore, we acknowledge that while the results suggest Nrf2 pathway involvement, functional validation is needed to substantiate this interpretation.

Collectively, our findings indicate that LSI312A modulates dendritic cell activation and inflammatory signaling through suppression of NF-κB phosphorylation and potential enhancement of Nrf2 signaling. However, these conclusions are limited by the lack of nuclear fractionation assays, target gene expression analyses, and apoptosis functional assays. Furthermore, the absence of *in vivo* validation constrains the translational relevance of our study. Future investigations should focus on evaluating LSI312A in primary dendritic cells, assessing its effects on T cell activation in co-culture systems, and determining its therapeutic potential in animal models of inflammatory and autoimmune diseases.

In conclusion, this study provides the first evidence that LSI312A possesses immunomodulatory activity in dendritic cells, characterized by reduced antigen uptake, suppressed co-stimulatory molecule expression, and decreased production of pro-inflammatory mediators. By modulating key signaling pathways involved in inflammation and redox balance, LSI312A emerges as a promising candidate for further development as an anti-inflammatory agent. However, additional mechanistic validation and in vivo studies are necessary to fully elucidate its therapeutic potential and safety profile.
